# Comparison of Experimentally Determined Two-Dimensional Strain Fields and Mapped Ultrasonic Data Processed by Coda Wave Interferometry

**DOI:** 10.3390/s20144023

**Published:** 2020-07-20

**Authors:** Felix Clauß, Niklas Epple, Mark Alexander Ahrens, Ernst Niederleithinger, Peter Mark

**Affiliations:** 1 Lehrstuhl für Massivbau, Fakultät für Bau- und Umweltingenieurwissenschaften, Ruhr-Universität Bochum, Universitätstraße 150, 44801 Bochum, Germany; Alexander.Ahrens@rub.de (M.A.A.); Peter.Mark@rub.de (P.M.); 2 Bundesanstalt für Materialforschung und -prüfung (BAM), Unter den Eichen 87, 12205 Berlin, Germany; Niklas.Epple@bam.de (N.E.); Ernst.Niederleithinger@bam.de (E.N.)

**Keywords:** digital image correlation, fiber optic sensors, stress, strain, Coda Wave Interferometry, ultrasound, concrete, tests, damage, crack

## Abstract

Due to the high sensitivity of coda waves to the smallest structural alterations such as strain, humidity or temperature changes, ultrasonic waves are a valid means to examine entire structures employing networks of ultrasonic transducers. In order to substantiate this ex ante assessment, the viability of measuring ultrasonic waves as a valid point of reference and inference for structural changes is to be further scrutinized in this work. In order to investigate the influence of mechanical strain on ultrasonic signals, a four-point bending test was carried out on a reinforced concrete beam at Ruhr University Bochum. Thus, measurements collected from a network of selected transducer pairings arranged across the central, shear-free segment of the test specimen, were correlated to their respective strain fields. Detected ultrasonic signals were evaluated employing Coda Wave Interferometry. Such analysis comprised the initial non-cracked state as well as later stages with incremental crack depth and quantity. It was to ascertain that the test specimen can in fact be qualitatively compartmentalized into areas of compression and tension identified via Relative Velocity Changes presented in Attribute Maps. However, since results did not entail a zero crossing, i.e., neither positive nor negative values were to be calculated, only relative changes in this work displayed staggered over the height of the object under test, are discussed. Under the given methodological premises, additional information is currently required to make quantitative assertions regarding this correlation of ultrasonic and strain results. This holds true for the comparability of the ultrasonic and strain results for both non-cracked and even the cracked state.

## 1. Introduction

With constantly growing traffic volumes, especially pertaining to cargo and heavy goods transport, putting increasing stress levels on an aging stock of transportation infrastructure (e.g., bridges [1,2,3]) and various changes in current standardizations looming large, calls for the development of a simple and comprehensive monitoring system are growing louder. Such a system, which must be designed for both new and existing structures, therefore needs to be able to carry out real-time damage assessments [4] in order to inform better-advised decision-making by public officials, for example, commissioning necessary strengthening of decaying structures. Ultrasound, as a signal that propagates through significant volumes or even entire structural elements, ushers in the possibility of achieving spatial results with a limited number of sources and receivers making it a potentially viable foundation for designing such versatile monitoring systems.

Ultrasonic (US) transmission measurements are in common use for determining local defects, for the assessment of freeze-thaw resistance, in concrete fatigue tests and even in monitoring load tests of pre-stressed bridge girders [5]. The assessment of freeze-thaw resistance is a common example for the evaluation of the direct wave (time of flight). By evaluating not only the first wave but also later arrivals—the coda—it is possible to take parts of the US signal under consideration that have interacted with larger areas of the concrete structure.

Because of inhomogeneities on the micro- and meso-level of concrete—like aggregates, pores or (micro-) cracks—the US signal is scattered multiple times and the wave is deflected from the direct path. By not only passing this direct path between a source and receiver, the coda contains information about an array of undergone volumes. It has been shown that stress, temperature [6,7] and moisture modifications as well as (micro-) cracks affect the coda signal. Due to the aforementioned sensitivity of US signals to small changes in the structure to be examined, it is possible to investigate the relation between these deviations on the one, and variation of the latter segment of the signal, i.e., the coda, on the other hand. Based on these results, real time assessments [6,8,9] of civil engineering structures seem possible. Depending on the geometric spreading of the US signal, it may enable not only real time assessment of structures but even early detection of possible future weak points and determination of areas where maintenance is required.

Recently, Coda Wave Interferometry (CWI) [10], a technique originating from the field of seismology, was also applied to civil engineering problems. It is based on the comparison of a reference signal and a signal including a modification of one or multiple of the aforementioned factors. By using a two-dimensional network of transducers (in this context, devices that are able to both send and receive ultrasound), it is possible to aggregate single results and map them as plane fields (in the following, used interchangeably with areas or zones indicating the two-dimensionality of data).

Beyond strain gauges, other measurement techniques, such as Digital Image Correlation (DIC) or Fiber Optic Sensors (FOS) have emerged in recent years. These offer the opportunity of not only measuring strains at one specific point, but also of investigating overall structural behavior via more encompassing field-like zones. However, the characteristics of concrete—for example, little tensile strength and the emergence of cracks—complicate the evaluation further. Nonetheless, there are recent studies [11,12] on calculating the crack width by integrating measured strains from FOS. Other research [13] has shown another advantage of this novel measuring technique so that the triaxial behavior of concrete can be further scrutinized via an adequate application of FOS. DIC, generating and contrasting photos of two-dimensional character, initially produces data on deformation fields, which subsequently provide the basis for computing strain fields. In contrast to these results, strains detected via FOS are initially measured solely along the fiber itself and therefore have to be pre-processed in order to generate areal, plane data for distinguishing between zones of, for example, compression or tension.

In this work, an experiment on a reinforced concrete (RC) beam subjected to four-point bending is presented. In addition to a network of US transducers, the test specimen was equipped with different, aforementioned techniques for strain measurement. Hereby, the focus lies on results produced by DIC and FOS. The calculated two-dimensional strain fields will be compared to mapped US data. For this purpose, CWI methodology was applied with a stepwise reference.

## 2. Principles of Measuring Methods

### 2.1. Strain Measurements

#### 2.1.1. Fiber Optic Sensors

Fiber optic devices enable detection of strain or temperature changes by evaluating the backscattering of an induced light beam in the fiber under test. Apart from the Rayleigh component of the backscattering, one can distinguish between Brillouin and Raman backscattering. As the fiber optic device employed here utilizes Rayleigh backscattering, further explanations will here be limited to this very component.

**Figure 1:**
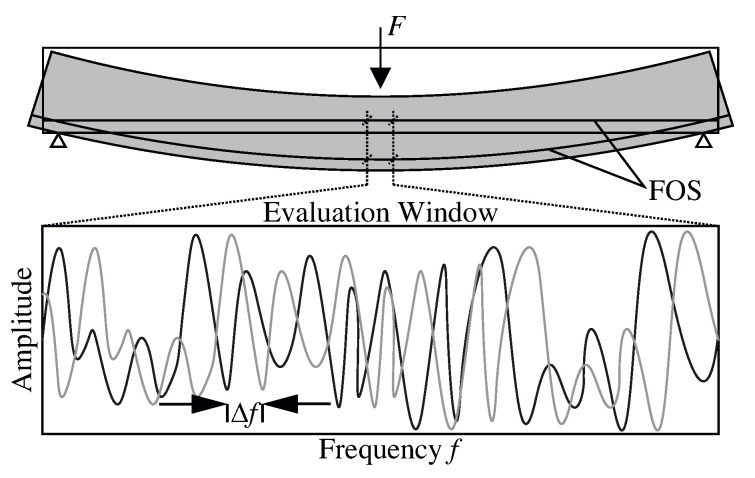
Frequency shift in the evaluation window caused by an exemplary loading.

Rayleigh backscattering can be attributed to quasi-stationary entropy fluctuations of anisotropic molecules [14]. Thus, when a light beam is emitted into a glass fiber, Rayleigh backscattering, which is hence caused by the variable refractive index along the fiber, is recorded by detectors in the fiber optic device. This measurement is done once in a reference state, such as the unloaded state, and during loading as shown in Figure 1. Two emerging signals result from this procedure. By performing a Fast Fourier Transformation, the two signals are converted into a frequency domain and afterward evaluated in smaller windows. The frequency shift Δf in an evaluation window can be related directly to the change in strain Δε and simultaneously in temperature ΔT, employing the coefficient of expansion $K_{\varepsilon }$ and temperature $K_{T}$, respectively, as well as the center wavelength λ and *c* the speed of light [15].

(1) $$\Delta \varepsilon \cdot \left[-\frac{c\cdot K_{\varepsilon }}{\lambda }\right]+\Delta T\cdot \left[-\frac{c\cdot K_{T}}{\lambda }\right]=\Delta f$$

As stated and depicted in Equation (1), frequency shifts are caused by a change in temperature as well as in strain. Therefore, one of these effects has to remain constant or has to be controlled by a second measurement. The values of the coefficients $K_{\varepsilon }$ and $K_{T}$ depend on the doping level of the fiber core as well as, to a lesser degree, on the composition of the cladding and coating [15]. The strain coefficient is, by a power of ten, greater than the temperature coefficient. This demonstrates that a small temperature change during a strain measurement is of minor importance. Conversely, a change in strain during a temperature measurement can lead to skewed results. It is here to be noted that the approach using a linear relationship between temperature and strain is simplified and should only be used for pure strain measurements. For real temperature measurements, a quartic polynomial is preferable, to be chosen according to [16]. Further sophisticated remarks on the application of FOS to test specimens can be found in [17].

#### 2.1.2. Digital Image Correlation

**Figure 2:**
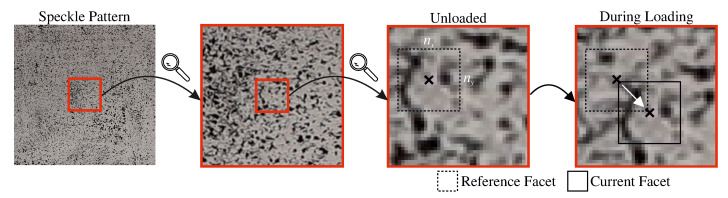
Speckle pattern and schematic representation of facet matching.

With DIC, surface strains can be calculated from detailed photos of a test specimen. Hence, a preferentially random speckle pattern is sprayed on the surface of interest. As shown in Figure 2, the speckle pattern is then divided into squares (i.e., facets) on the pixel level. These facets are the basis for the calculation of displacements (at each facet) and strains for a meta area. Dividing the speckle pattern into facets results in matrices consisting of $n_{x}\times n_{y}$ gray tones. Thus, a unique set of gray tones is assigned to the center of each facet. After the test specimen is deformed by an applied load, the facet experiences movement from the position it held prior to the application of loading—it is moved from its initial position. The aim is to retrieve the reference set of gray tones (reference facet) from the unloaded state, in the current, post-load image. During loading, each facet, and therefore its individual set of gray tones, can be shifted, rotated and slightly changed by modified light exposure at its updated position. For purposes of reducing complexity, however, the facet depicted in Figure 2 is only shifted [18].

In facet matching, various factors such as shifting, rotation and perspective distortion of the facet itself or possible contrast and lightning changes of the facet in its updated, potentially altered position have to be taken into consideration. Therefore the gray tone matrix of the reference facet $g_{M}\left(x_{i},y_{i}\right)$ and a search matrix $g_{Si}\left(x_{it},y_{it}\right)$, which contains the current (loaded/transformed) facet, are interpolated to determine deformations smaller than one pixel. The application of the least squares method to these matrices leads to Equation (2) [18,19]. The coefficients $r_{0}$ and $r_{1}$ describe the relative camera offset and relative camera gain, respectively.

(2) $$\min\sum_{i=1}^{n}{\left|g_{M}\left(x_{i},y_{i}\right)-\left(r_{0}+r_{1}\cdot g_{Si}\left(x_{it},y_{it}\right)\right)\right|}^{2}$$

Facet matching determines the displacement of each facet center. Using the deformation results from the direct vicinity of each point, strain can be calculated for a meta area.

### 2.2. Ultrasound

**Figure 3:**
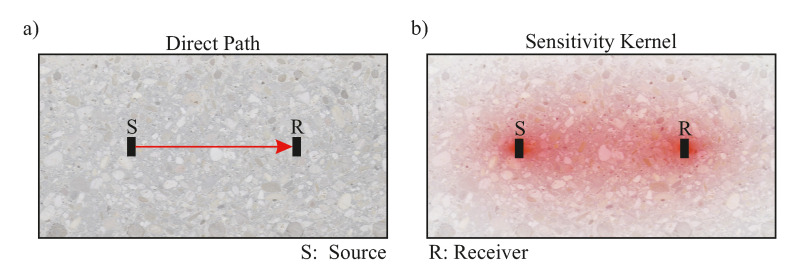
In classical ultrasonic (US) measurements, travel times are recorded and calculated via the ballistic waves. (**a**) Ballistic wave between source and receiver. Using coda waves, changes of travel time within a larger segment, described by (**b**) the sensitivity kernel (red), can be resolved.

The wave speed of an acoustic wave is an inherent property of the medium. While under constant conditions, wave speed—and in fact the entire waveform—of a repeated US measurement should not change at all, changes in the medium, for example, compression, tension or cracking, will however influence parameters like velocity, phase or attenuation. To detect these changes, most state-of-the-art US Non-Destructive Testing (NDT) methods use the first arrival of the direct, ballistic waves between source and receiver. In contrast, CWI uses the later segments, i.e., the coda, of the US recording to determine differences between repeated measurements and calculate velocity changes. Multiply scattered coda waves spend more time in the medium and sense a larger area before they are recorded at the receiver. Such behavior is illustrated in Figure 3. While the first, ballistic waves only record changes on the direct path, coda waves have sensed a wider area by the time they reach the receiver. This area—or the area a measurement is sensitive to—can be described by the sensitivity kernel for all times $t_{n}$ after the source excitation [20]. Therefore, subtle changes in the sensitive zones between and around source and receiver, which would not influence the direct wave investigated in standard NDT-US measurements, can be captured.

**Figure 4:**
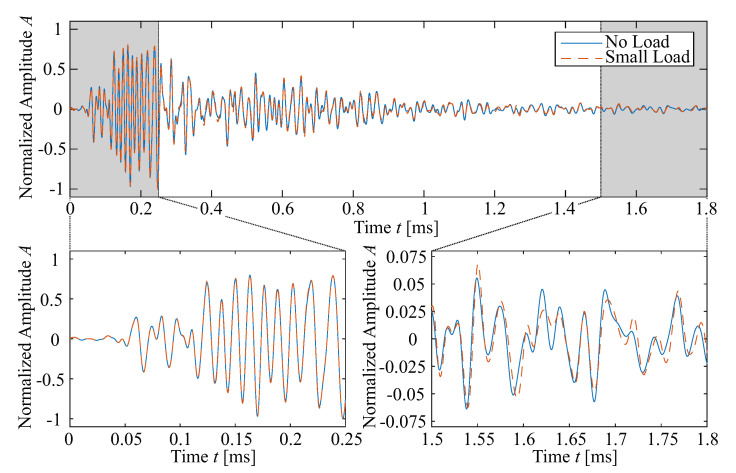
A comparison of measurements with embedded transducers in a concrete beam shows the small influence of subtle changes. While in the first arrivals (bottom left) no change is visible, small differences in the later coda part indicate a change in the specimen (bottom right).

In Figure 4, the influence of a small load on an US measurement using embedded transducers in a concrete beam is demonstrated. While there is no obvious change visible when looking at the entire recording and while the first arrival and early section of the recording between 0 and 0.25 ms are not changing, a shift of peaks and troughs can be discerned in the coda.

This property is used in CWI to quantify changes in the sensed medium. The technique is based on a comparison of two recordings between the same sources and receivers at different times. As a measure for similarity between waveforms, the Correlation Coefficient (CC) is commonly used as follows:

(3) $$CC\left(t\right)=\frac{\int_{T}u_{u}\left(t\right)u_{pt}\left(t\right)dt}{\sqrt{\int_{T}u_{u}^{2}\left(t\right)\int_{T}u_{pt}^{2}\left(t\right)dt}}$$

The unperturbed wave field $u_{u}$ and the perturbed wave field $u_{pt}$ are compared on a time interval *T*, where the resulting coefficient is {CC|−1≤CC≤1}. If $u_{u}$ and $u_{pt}$ are similar, CC will be close to 1. If they are similar but the phase is shifted by 180 degrees, it is close to −1. In the case of completely different waveforms, the coefficient is zero. As the CC is only a measure of similarity, velocity changes need to be calculated with more advanced operations. To such ends, two techniques for the determination of velocity changes are commonly applied. The first one, proposed in [21,22,23] compares the signals in small time windows and determines the time shift maximizing the CC. This time shift is linked to a Relative Velocity Change (dt/t=−dv/v). The second technique, the so-called stretching technique introduced in [24,25,26], utilizes the larger parts of the coda on the interval between $t_{1}$ and $t_{2}$ to determine a stretching factor α=−dv/v rather than a time shift. Therefore, the unperturbed signal is stretched by α, which maximizes the CC.

(4) $$CC\left(t,\alpha \right)=\frac{\int_{t_{1}}^{t_{2}}u_{u}\left(t+\alpha \right)u_{pt}\left(t\right)dt}{\sqrt{\int_{t_{1}}^{t_{2}}u_{u}^{2}\left(t+\alpha \right)\int_{t_{1}}^{t_{2}}u_{pt}^{2}\left(t\right)dt}}$$

If only the overall velocity of propagation of the sensed medium changes, the US signal recorded after this change is a stretched (or compressed) copy of the original signal. Just the mean velocity but not the scattering properties changes. The α maximizing Equation (4) can then be linked to the apparent overall velocity change in the sensed medium. However, there is no direct link to a physical property. Since the latter technique has both produced robust results and been applied frequently in recent years ([5]), it will be applied in the analysis of the US measurements in this paper. In a monitoring set-up, where many consecutive measurements are evaluated, a reference measurement needs to be chosen. This reference can be static, for example, choosing the first measurement as the zero-state. dv/v and CC are then calculated with respect to this Fixed-Reference CWI signal. Alternatively (discussed in [5,27]), the reference signal can also be changed in a stepwise fashion. This ensures a strong similarity of the compared signals—if no major damages occur—and is thus helpful in long-term experiments, as well as in experiments where the changes to the material are substantial and even destructive. While the fixed-reference method is computationally less expensive, it will fail to produce good results as to long-ranging experiments or experiments where the specimen is destroyed in many cases. If the correlation between two measurements is too small (<0.7), the calculated dv/v will be unstable and have to be interpreted with caution. The dv/v calculated in the stepwise procedure can be accumulated and linked to the zero-state. A calculation of the total velocity changes throughout the whole experiment is possible, while the CC and dv/v calculations remain stable.

## 3. Experiments

### 3.1. Method of Investigation

As shown in Figure 3, the coda part of the US signal consists of information accumulated from sensing a larger undergone area. In the aforementioned CWI, an evaluation window is defined for which CC and dv/v are calculated. By choosing a relatively long evaluation window, for example, from 0.75 ms to 1.8 ms (cf. Figure 4), the results of the CWI are calculated as an integral over the full undergone volume.

In order to compare strain fields and US maps and with respect to the integration of results over larger areas, it is advisable to investigate an area with constant strains. Therefore, an RC beam subjected to four-point bending was determined as an appropriate testing specimen. Resulting from such a load scenario, a constant bending moment and thus likewise a constant strain state between the concentrated loads can be generated. Nevertheless, the general difference between static systems and real test set-ups likewise applies to the experiment at hand. Thus, in practice, there cannot be an ideal form of concentrated load induction for the areas underneath. On these grounds the transducers are placed at a distance of 130 mm in order to avoid said area, i.e., the area between an associated source and receiver pair. Furthermore, only the US transducers located between the two concentrated loads are selected and considered in later evaluations.

### 3.2. Test Set-Up

For the execution of such tests, an RC beam was cast at Ruhr University Bochum. Said beam was designed as to the dimensions of 150 mm/400 mm/2000 mm (*b*/*h*/$l_{eff}$), specifically suiting four-point bending. This entails a bending reinforcement of (2∅16 mm) and stirrups (∅10 mm/20 cm/2). An additional constructive reinforcement (2∅8 mm) was arranged in the compression zone. For simplified attachment of US transducers, the stirrups were also arranged in the shear-free zone between the two concentrated loads. The beam was concreted on the side surface, implying multiple benefits. Most notably, such a casting process generated a smooth surface for the DIC, allowed for a simplified handling of all sensors during concreting and contributed to better compaction of the test specimen. In addition to the test specimen, various concrete cubes and cylinders were made to determine the material properties in accompanying tests. All relevant parameters after 28 days of curing are summarized in Table 1.

**Figure 5:**
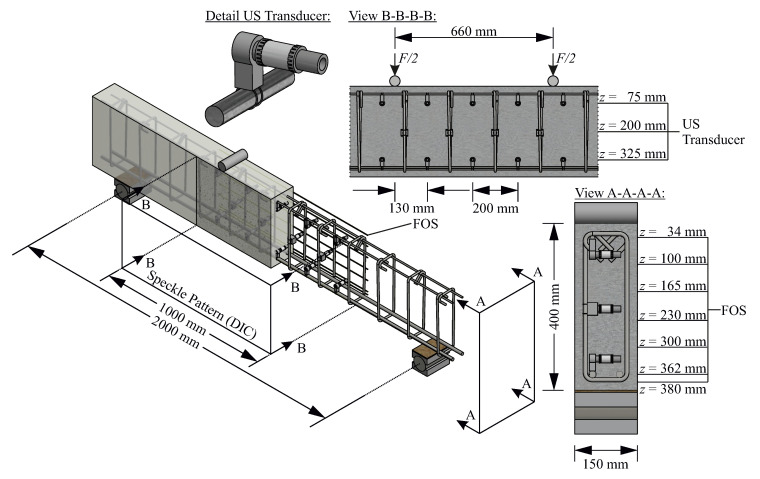
Model of the test specimen.

In total, eight FOS were placed within the object under test. While one was applied on the bending reinforcement in a notch along the bar, the remaining sensors were attached on the side surface of the RC beam. Along with placements at characteristic heights, such as the upper and lower reinforcement, the remaining FOS were allocated staggered with equal clearance over the beam’s height. Their precise positioning is to be found in Figure 5. Said FOS were glued onto the reinforcement steel as well as on the concrete utilizing a special epoxy resin. This particular adhesive had been examined in earlier experiments. It had thus become evident that this particular epoxy resin was indeed stiff enough to transfer the strain from concrete or steel directly into the fiber, while simultaneously displaying the benefit of smoothening the otherwise highly fluctuating measured strains.

**Figure 6:**
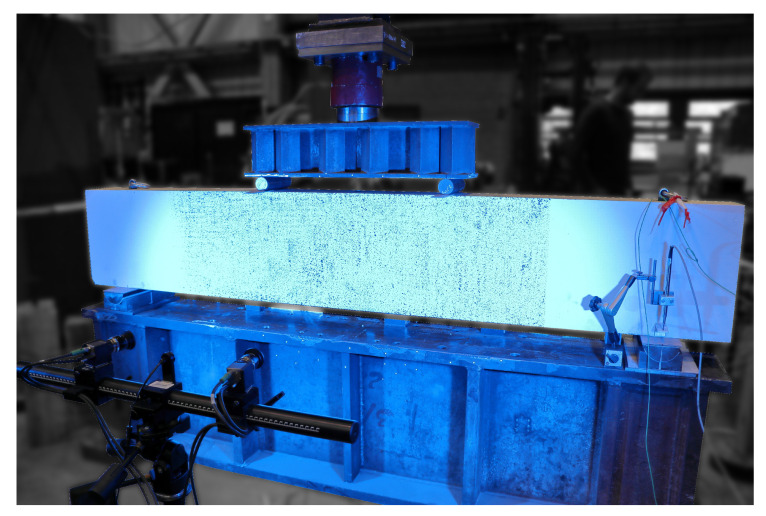
Photo of the test set-up.

An addition to the test beam consisted of initially applying white paint to a predetermined area, which, upon subsequently adding a black speckle pattern, was to enable DIC (a field of 1000mm×400mm=b×h) in latter stages of the experiment. For accuracy reasons, the two cameras were placed in immediate proximity (cf. Figure 6), as close as possible to the specimen in order to maximize pixel density.

Besides these strain sensors, US transducers were arranged in the central area of the beam forming a network structure. SO807 transducers from Acoustic Control Systems, Ltd. (Sarrebruck, Germany), with a central frequency of 60 kHz, are utilized. They consist of a piezoceramic cylinder with a diameter of 20 mm and a length of 35 mm. The radiation is characterized in [28], where it is shown that the US signal is emitted almost uniform in all directions of space. By employing small cement clips to position said transducers, it was to be ensured that their direct surrounding was constituted by largely homogeneous material. Such clips, in contrast to plastic attachments, for example, exert minimal effect on wave propagation and crack formation during loading. The US transducers were thus in three levels arranged over the height of the RC beam. For the imaging to be applied later, 14 of them were allocated in the middle area of the beam. The transducers of the upper and lower level were attached to the bending or constructive reinforcement, respectively. Precisely between these two, the transducers of the central level were attached to the stirrups. By prior finite element analysis, regions were identified below the steel cylinders (employed to induce the concentrated loads in four-point bending—see Figure 5) where the principal stresses in the shear-free, central region are inclined (not horizontally orientated). Here, the stirrups and, in particular, the transducers of the upper layer were shifted inwards by 130 mm. Consequently, no transducers were placed directly below the concentrated loads or in the region affected by load introduction. Thus, the affected region does not overlap the region of the eight central transducers. Nevertheless, measurements through the affected region were performed, too, but are excluded from analysis since they exceed the scope of the recent work.

Loading of the beam progressed in an incremental fashion. Furthermore, on each of the load levels, all types of measurement, i.e., FOS, DIC, US, were performed simultaneously. The US measurements were carried out consecutively between all adjacent pairs of transducers, i.e., for each source-receiver combination, one transducer emits an US signal while the other picks up this signal. Successive switching of all adjacent combinations yields 32 individual measurements between the sensor pairs per load level. Simultaneous measurements are performed with the FOS as well as with the DIC. This synchronicity results in consistency across said techniques.

Moreover, the ambient temperature in the laboratory was monitored during testing. Taking this into account, the minimal changes in temperature present at the actual testing site were not expected to have any influence on the different measuring methodologies. Mainly due to the relatively low thermal conductivity of approx. 2 W/(m·K), the heat transfer in concrete is quite inert. During the experiment, the ambient temperature change was <0.5 ${}^{\circ }$C. Thus, it is assumed that the spatial temperature variation in the concrete is negligible, which is also confirmed by the temperature measurements.

The investigations in the further course of this project and the associated findings are deemed to enable to lighten the yet quite fine network of transducers (maintaining the high density of results of the CWI) and thus to justify practical application to real structures such as bridges.

### 3.3. Results

#### 3.3.1. Strain

**Figure 7:**
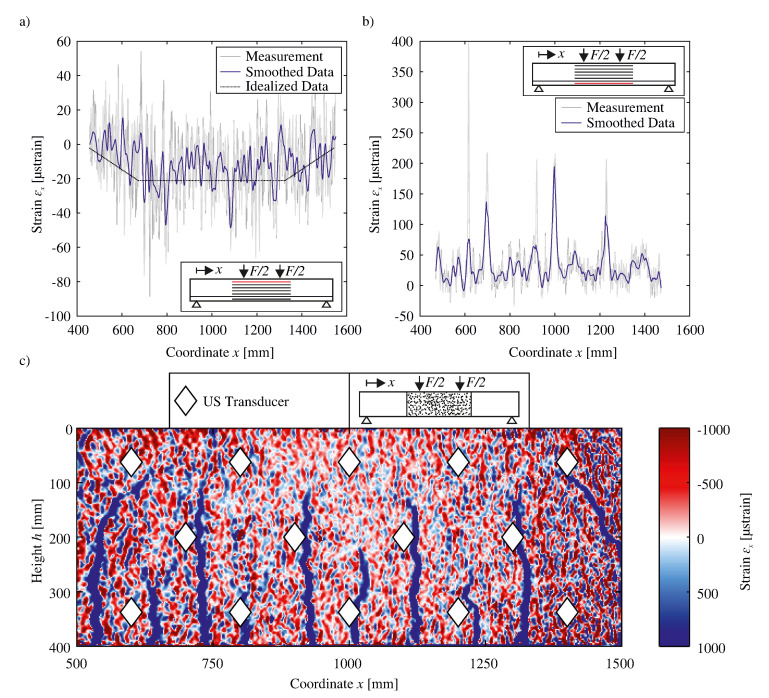
(**a**,**b**) Strain from Fiber Optic Sensors (FOS) for an applied load of F=10kN and (**c**) strain from Digital Image Correlation (DIC) for an applied load of F=160kN and marked US transducer positions.

Due to fiber optic technology’s general measuring characteristics, a quasi-continuous strain curve is recorded during the experiment. Here, the following mode of operation was selected: length of the evaluation window of 1.3 mm and thus point distance of 0.65 mm. Figure 7a depicts the compressive strain measured by an FOS. The FOS is located 34 mm underneath the top of the RC beam (also see Figure 5). The unprocessed strains are displayed in gray. Both due to the macroscopic differing Young’s modulus of the concrete as well as the measurement noise, highly fluctuating strains are measured. In order to improve their interpretability, the original measured data are smoothed with a piecewise robust regression. As Figure 7a displays, the original data (Measurement) are partly in the positive range, although this FOS is allocated in the compression zone. This observation can be traced back to the measuring noise, which, in relative terms, has a stronger impact during lower loads. Symmetrically originating at both ends, compressive strains are to be observed. Following the constant bending moment between the two concentrated loads, a constant strain level between x≈700mm and x≈1300mm is highlighted (cf. dotted line in Figure 7a).

Analogously, Figure 7b illustrates the measured strains for a FOS. However, in contrast to before, this sensor is located in the tensile zone at a distance of 380 mm to the top of the beam. Estimating the concrete strain at the formation of the first crack at 0.1‰ ≡100μstrain, leads to the safe assumption that initial cracks have already emerged. Due to the attachment of the FOS to the concrete surface, the relative shift of the crack edges leads to increasing strains measured at the precise position of a crack. Nevertheless, it is here to be constituted that decreasing stresses and thus also strains towards the crack edges cannot be measured this way.

Figure 7c shows the two-dimensional strain field measured by DIC. The underlying displacements used to calculate these strains are estimated for a facet of 19 × 19 pixels. The measured deformation in a specific facet center (i.e., its displacement) allows for a careful calculation and deduction of strain for meta areas by aggregating the deformation of said facet center itself with deformation data from facet centers in its direct vicinity. Due to this summarizing process, the resolution of DIC strain results suffers, for example, leading to an overemphasis of crack width, as is evident in Figure 7c. Furthermore, it is pointed out that, besides the cracks themselves (displayed in continuous blue lines), no strains can be identified because of the strong measuring noise. From the extensive white areas between x≈800mm and x≈1200mm, it is to be derived that this area exhibits low measurement noise. This can be traced back to the unavoidable picture blur of the image at the horizontal edges of the image. Figure 7c displays a regular crack pattern. Two flexural-shear cracks, one on each side, four primary flexural cracks and four secondary flexural cracks in-between can be identified. A more detailed juxtaposition of the crack pattern and the US transducer positions shows that in most cases the cracks did not propagate into the transducers. Therefore, it is to be inferred that in fact no uncoupling of any transducers has occurred. In short, crack propagation and thus the resulting crack pattern is not affected by the transducers, thereby demonstrating that said transducers do not significantly impact load bearing behavior.

The occurrence as well as the propagation of secondary flexural cracks in this experiment are confirmed by general model representations in extant literature. Said models localize secondary flexural cracking in detail. Projecting the point of intersection of the bending reinforcement and a primary flexural crack at an angle of 45 degrees, such projection intersects the upper tip of a secondary flexural crack (cf. [29]).

#### 3.3.2. Ultrasound

**Figure 8:**
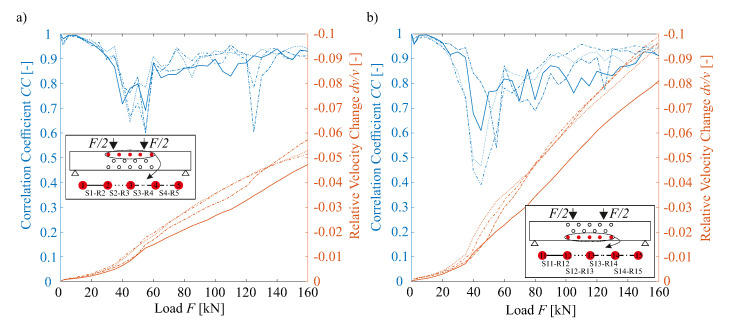
Stepwise Correlation Coefficient CC and cumulative Relative Velocity Change dv/v for selected transducer pairs in the (**a**) compressive and (**b**) tensile zone.

On each load level, measurements of all transducer combinations have been carried out. Figure 8 illustrates the CC and dv/v calculated with the Stepwise CWI. Figure 8a,b detail the results of the CWI for exemplary transducer pairs in the compressive (Figure 8a) and tensile zone (Figure 8b), respectively. As is evident (in Figure 8), the CC decreases up to a load of approximately F=40 kN to 55 kN. This global minimum can be confirmed for every transducer pair. The CC for transducer pairs in the compressive zone decreases to the range [0.60, 0.70], while the one for pairs in tension yields [0.40, 0.60]. Due to micro-cracking in the tensile zone, the decorrelation (1−CC) is more pronounced here.

Based on the tensile strength of the concrete ($f_{ctm}=2.5\;N/{\mathrm{mm}}^{2}$) and according to the dimensions of the static system, 30 kN constitutes the level of force required to create the first crack. By applying a coefficient of variation of 0.3, i.e., concrete’s tensile strength, a completed crack pattern can be expected to emerge at a force level of around 40 kN (cf. [29,30]). Such theoretical considerations can here be confirmed employing the strain measurement results of the fiber optics. Therefore, it can be concluded that the initial formation of cracks has a higher influence on the correlation than the subsequent crack expansion. Moreover, in contrast to the aforementioned Fixed-Reference CWI, the CC calculated via Stepwise CWI is in fact able to recover from and even increase after a temporary decline.

Within a range of approximately F=40 kN to 160 kN, multiple local minimums of the CC are identifiable. Furthermore, it can be observed for both cases that the CC decreases by several percent right at the beginning. In this context, such CC behavior is largely attributable to the relocation from a continuous supporting on elastomer bearings to the supporting in the four-point bending test set-up right before the test.

Because the dv/v in Stepwise CWI is only calculated with respect to the previous US measurement, results need to be accumulated. Said fact constitutes the reason why the dv/v continuously increases during loading. It is to be noted that the gradients of the transducer pairs in the compressive as well as in the tensile zone increase after a load of F=40 kN. Furthermore, it can be ascertained that a smaller dv/v is calculated in the compressive than in the tensile zone.

## 4. Comparison of US Results and Strain Fields

### 4.1. Non-Cracked to Slightly Cracked State

**Figure 9:**
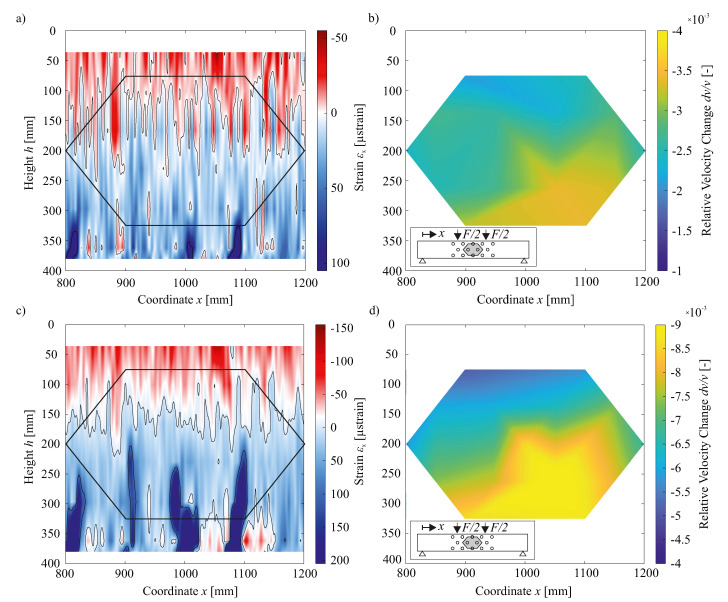
(**a**,**c**) Strain fields derived by FOS measurements assigned to their respective heights, (**b**,**d**) Relative Velocity Change presented as Attribute Maps. The two upper figures show the respective results for a load of F=10 kN, while the two lower Figures do so for F=25 kN.

As previously discussed, the FOS were attached onto the concrete beam, staggered in equal clearance over its height. The measurement output of one fiber consists of one-dimensional strain data with a point distance of 0.65 mm. Assigning the strain from multiple FOS to their respective positionings and heights on the RC beam, allows for an aggregation of the one-dimensional strain data points forming two-dimensional strain fields. Figure 9a,c depict such mapped strain data. Values in-between the support lines (FOS data with given height) were linearly interpolated. Since no extrapolation is carried out, the outer FOS (at a height of 34 and 380 mm) represent the limits of the strain field.

Figure 9b,d show the dv/v presented as a two-dimensional map (Attribute Map, cf. [31]). The solitary results of each transducer pair were located right in the middle between them and intermediate values were linearly interpolated. Positioning the results in the middle of two transducer pairs leads to an Attribute Map with the y-limits of 75 and 325 mm. Note that the color scale (e.g., limits of dv/v) differs for the subsequent representation of Attribute Maps due to the large variation of dv/v with raising load.

The juxtaposition of strain field and Attribute Map anew reiterates that the tensile zone shows larger dv/v than the compressive zone. When the test specimen at hand cracks, the height of said tensile zone approximately equals the depth of primary flexural cracks. Also, the enhanced right-sided crack occurrence is to be identified by the Attribute Map. A comparison of Figure 9b,d reveals how dv/v rises with increasing tensile strain and therefore likewise with growing load.

Figure 9a,b represents strain and US data for a load level of 10 kN. Because of the low strain values and the, in relative terms, high measuring noise, no definite compressive zone can be identified in Figure 9a. However, the height of the compressive zone at a load of 10 kN is estimated to range between h=200 mm and h=150 mm. Furthermore, in the tensile zone, at x≈ 800 mm/1000 mm/1100 mm, small strain peaks are visible, reaching approximately 100μstrain (noted earlier as the approximate strain level at which concrete first cracks). Further scrutiny of the coordinates of said strain peaks again emphasizes that higher tensile strain is present on the beam’s right side.

Figure 9b demonstrates greater dv/v present in the lower part of the test specimen compared to the upper part. The dv/v ranges from approximately $-2.25\cdot 10^{-3}$ in the compressive to $-4\cdot 10^{-3}$ in the tensile zone of the beam. In addition, a right-sided concentration of the largest dv/v can be observed. Due to the missing zero crossing of the dv/v, measured results can only be interpreted in relation to one another, i.e., as relative change over height. In order to be able to deduce the strain state from the Attribute Maps, additional information, such as the type of external force, is required. Based on a juxtaposition of strain fields and Attribute Maps, later analyses of data presented in the Attribute Maps allow for distinguishing tensile from compressive strains present in the beam. Thus, in relative terms, tensile strains overall cause a greater change in dv/v than compressive strains. The aforementioned clustering of strains on the right side, is likewise underpinned by the US results. As discussed earlier, Figure 9c,d exhibits the strain and Attribute Map for a load of 25 kN. Both depictions point out that, with increasing load and the associated increase in strain, measurement noise plays a lessened role compared to the lower strain levels presented earlier (cf. Figure 9a). Thus, such relatively low measurement noise at higher strain levels renders it possible to approximate the compression zone height more precisely as lying between 150 and 175 mm. Furthermore, in addition to the points of increased strain from Figure 9a, the higher strain level presented in Figure 9c also features further areas of increased strain, which continue to expand vertically in a linear manner. The structure of results for the zones in which increased strain of over +100μstrain occurs, indicates concrete cracking in these areas. It should be noted that strains exceeding +100μstrain are only measured due to the crack edges moving relative to one another (cf. Figure 7). In said cracked sections, dv/v analogously also increases, as evident in the strain field. The previously identified phenomenon of tensile zones showing larger dv/v than compressive zones, can also be deduced from Figure 9d, so that there are values ranging from $-6\cdot 10^{-3}$ to $-9\cdot 10^{-3}$. In accordance with previous results highlighting the right-sided clustering of higher strains, dv/v likewise dominate in this area of the beam.

### 4.2. Completed Crack Pattern and Increasing Crack Widening

In contrast to Figure 9, the strain fields are here measured utilizing DIC. Therefore, the measured strain data is presented from h=25 mm to 375 mm. Depending on the rigidity of the epoxy resin adhesive needed to attach the FOS to the rebar or concrete surface, the conjunction of FOS and resin is susceptible to generating slightly skewed results. Here, high strain areas affect FOS measurements in its surroundings, as strain is diverted due to the (inevitably) limited rigidity of the resin. Due to such limitations of FOS-based strain measurements, DIC serves as a vital complement.

**Figure 10:**
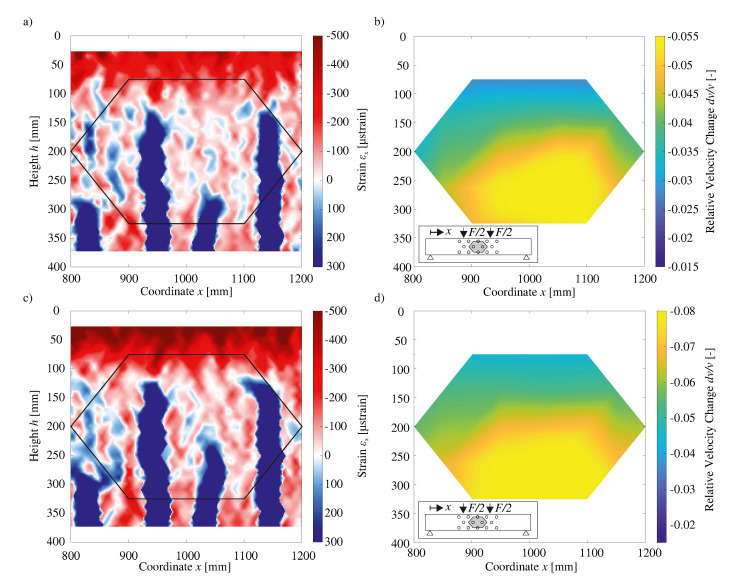
(**a**,**c**) Strain fields from DIC, (**b**,**d**) Relative Velocity Changes dv/v presented as Attribute Maps. Figures above show results for a load of F=75 kN, Figures below for F=120 kN, respectively.

In order to calculate strains, DIC measures deformations employing facet sizes of 49 × 49 pixels as exemplified in Figure 10. Due to such resolution, the portrayal of the size of individual cracks (blue line-like strains) appears to be enlarged when depicted in Figure 10. By using additional pixels for a facet, additional information is available to the algorithm, which, in turn, is able to determine the displacement of a facet during loading more accurately. Moreover, such an addition (of pixels) encompasses a larger area being available for calculating a meta area’s strain levels, so that strain overall can be calculated more meticulously. Thus, notwithstanding the production of more accurate strain results, the density of the results is reduced following this method.

Figure 10b,d shows an Attribute Map for the dv/v measured by the eight centrally located US transducers. It is made evident that the qualitative appearance of the Attribute Map is similar to that of Figure 9b,d, whereas only the magnitude of the dv/v differs. As previously discussed, the right-sided concentration of cracking here again finds support in the Attribute Maps. Consequently, it is to be inferred that even an augmented crack propagation does not hinder the evaluation of the US results. As annotated earlier, the tensile strength of the used concrete requires a force of 30 kN, in order to engender initial cracking. Nevertheless, as Figure 7 emphasized, strain peaks, from which cracking is to be expected, can be detected much earlier than 30 kN. Similar to the previous illustration in Figure 9, a strain field and an Attribute Map for a load of 75 kN (Figure 10a,b) and 120 kN (Figure 10c,d) are displayed. The comparison of Figure 10a,c underlines that the strain in the compression zone rises with increasing load. At the same time, the primary flexural cracks propagate only slightly. Due to the progressive crack growth, a compression zone height of approximately 100 to 125 mm can thus be determined for both loads shown here. Due to the high measurement noise present, the low strain zones between cracks cannot be assessed regarding strain distribution.

## 5. Discussion and Conclusions

In this paper, experiments pertaining to US measuring techniques for the monitoring of large concrete structures performed on an RC beam subjected to four-point bending were presented. To such ends, the test specimen was equipped with a network of US transducers in addition to FOS and DIC systems. In contrast to the results of the DIC, the measurement data of individual FOS must initially be aggregated in order to calculate strain fields. For this purpose, the individual measured values are assigned to the respective placement of the FOS on the test beam and can thus be displayed as strain fields. The US results of respective source-receiver pairings must likewise undergo previous processing employing Stepwise CWI, before being assigned to a location along the test object. Finally, the illustration of dv/v in the form of Attribute Maps creates a representation resembling that of strain fields. A juxtaposition of strain fields and Attribute Maps eventuates in the understanding that US results can, in fact, serve to qualitatively identify compressive and tensile zones.

Under the given premises, however, the applied evaluation method does not allow for drawing inferences as to a quantitative correlation between the US results and strain fields, derived by means of FOS and DIC data. Nevertheless, it is ascertained that the extent of dv/v grows with increasing load, therefore also strain. Moreover, compression and tension zones as well as locally concentrated cracking measured via US transducers, concurs with measured strain data, rendering it possible to qualitatively attest extensive compatibility of US data on the one hand and FOS and DIC data on the other hand. Consequently, Stepwise CWI is able to produce viable results even in spite of increased cracking. Moreover, the evaluation of individual source-receiver pairings has also underlined that even the smallest changes, for example, repositioning the beam from a continuous bearing on elastomers to a bearing in a four-point bending test, can be detected by the US signal. Strains caused by such changes are generally far below the sensitivity of conventional and even novel strain measurement techniques.

Furthermore, FOS results indicate that a mere third of the force, which is customarily deemed necessary to create cracks in concrete (i.e., 30 kN), suffices in practice to generate cracks in the test specimen. As discussed, dv/v measured via US transducers are present in concentrated fashion on the right-hand side akin to strain measured via FOS. Such parallel properties provide reason for assuming that the US signal detected for a force level starting at 10 kN also points toward early cracking. However, such damage cannot be detected through optical inspection.

Therefore, it is here to be concluded that the use of CWI for the early detection of damage to concrete structures has substantial potential for practice.

## Figures and Tables

**Table 1:** Material properties of the concrete.

$f_{\mathrm{ck},\mathrm{cube}}$	$f_{\mathrm{ctm}}$	$E_{\mathrm{cm}}$
$\left[N/{\mathrm{mm}}^{2}\right]$	$\left[N/{\mathrm{mm}}^{2}\right]$	$\left[N/{\mathrm{mm}}^{2}\right]$
35.0	2.5	28,618

## Abbreviations

The following abbreviations are used in this manuscript:

US	Ultrasonic
CWI	Code Wave Interferometry
DIC	Digital Image Correlation
FOS	Fiber Optic Sensor
RC	Reinforced Concrete
NDT	Non-Destructive Testing
